# Fabrication of CuYO_2_ Nanofibers by Electrospinning and Applied to Hydrogen Harvest

**DOI:** 10.3390/ma15248957

**Published:** 2022-12-15

**Authors:** Kai-Chun Hsu, Arjunan Karthi Keyan, Chin-Wei Hung, Subramanian Sakthinathan, Chung-Lun Yu, Te-Wei Chiu, Karuppiah Nagaraj, Fang-Yu Fan, Yung-Kang Shan, Po-Chou Chen

**Affiliations:** 1 Department of Materials and Mineral Resources Engineering, National Taipei University of Technology, No. 1, Section 3, Zhongxiao E. Rd., Taipei 106, Taiwan; 2 Institute of Materials Science and Engineering, National Taipei University of Technology, No. 1, Section 3, Chung-Hsiao East Road, Taipei 106, Taiwan; 3 School of Dental Technology, Taipei Medical University, 250, Wu-Hsing Street, Taipei 110, Taiwan; 4 SRICT-Institute of Science and Research, UPL University of Sustainable Technology, Block No. 402, Ankleshwar-Valia, Rd., Vataria, Ankleshwar 393135, Gujarat, India; 5 E-Current Co., Ltd., 10F.-5, No. 50, Sec. 4, Nanjing E. Rd., Songshan Dist., Taipei 10553, Taiwan

**Keywords:** delafossite, electrospinning, CuYO_2_ nanofibers, methanol steam reforming, nanofibers, hydrogen production

## Abstract

Hydrogen can be employed as an alternative renewable energy source in response to climate change, global warming, and the energy problem. Methanol gas steam reforming (SRM) is the major method used in industry to produce hydrogen. In the SRM process, the catalyst nature offers benefits such as low cost, simplicity, and quickness. In this work, delafossite copper yttrium oxide (CuYO_2_) nanofibers were successfully prepared by electrospinning. The prepared CuYO_2_ nanofibers have different physical and chemical properties including thermoelectric behavior. The electrospinning method was used to produce as-spun fibers and annealed in an air atmosphere to form Cu_2_Y_2_O_5_ fibers; then, Cu_2_Y_2_O_5_ fibers were annealed in a nitrogen atmosphere to form CuYO_2_ nanofibers. X-ray diffraction studies and thermogravimetric and transmission electron microscope analysis confirmed the formation of CuYO_2_ nanofibers. The CuYO_2_ nanofibers were applied to methanol steam reforming for hydrogen production to confirm their catalytic ability. The CuYO_2_ nanofibers exhibited high catalytic activity and the best hydrogen production rate of 1967.89 mL min^−1^ g-cat^−1^ at 500 °C. The highly specific surface area of CuYO_2_ nanofibers used in steam reforming reactions could have significant economic and industrial implications. The performance of these CuYO_2_ nanofibers in hydrogen generation could be very important in industries with a global economic impact. Furthermore, the H_2_ production performance increases at higher reaction temperatures.

## 1. Introduction

Delafossite oxides of Cu_I_M_III_O_2_ (M = (Al, Ga, Fe, Cr, Y …)) have a combination of the monovalent metal Cu_I_ and the trivalent metal M_III_. The structural component of delafossite is Cu_I_ and MO_6_ octahedron layers along the *c* axis. The upper and bottom oxygen of the MO_6_ octahedron is linearly coordinated to two Cu_I_ atoms [1,2,3,4]. The majority of research on optoelectronic properties, such as transparent semiconductors, dye-sensitized solar cells, thermoelectric properties, photo electrodes, luminescence, photo-catalysis, antibacterial, gas sensing, energy storage, and superconductivity properties, are also focused on delafossite materials based on their structural characteristics [5].

Copper complexes have been regarded as multifunctional materials because of their widespread industrial use as a chemical reaction catalyst for hydrogen synthesis, dehydrogenation, oxidation, and alkylation [6,7,8]. Copper yttrium oxide (CuYO_2_) nanofiber is a Cu-based delafossite that is now being studied because of its appealing physical characteristics for the aforementioned uses [9]. According to reports, the delafossite structure of CuYO_2_ has a p-type and wide-bandgap with a high See-beck coefficient of the semiconductor (+274 VK^−1^). The physical properties of CuYO_2_ in the a–b plane is compared to the c-axis direction. This suggests that the thermoelectric properties are fairly high. These CuYO_2_ metal oxide compounds are a good contender for high-temperature thermoelectric applications in air [10]. CuYO_2_ powder consistently exhibits excellent catalytic efficiency in a variety of reactions, including methanol steam reforming (MSR) and water–gas conversion [11].

This study applies the delafossite structure of a catalyst to methanol steam reforming for hydrogen production. CuYO_2_ nanofibers were synthesized as a catalyst. Sun et al. have reported that Y_2_O_3_ is helpful for alcohol dehydrogenation, as CuYO_2_ is reduced to Cu^+^Y_2_O_3_ after MSR, which was chosen for better catalytic effectiveness. Due to the reaction principle of the fixed-bed reactor, the solid catalyst or solid reactant is typically in the form of particles, and the fluid reacts by stacking in a bed of a certain thickness [12].

The electrospinning method creates fibers by stretching a liquid in an electric field using the self-repulsion effect. It causes an electrostatic charge on a precursor material. The electrospinning parameters, such as applied voltage, viscosity, and weight, have a significant impact on the morphology of the resulting fibers and can be adjusted for the desired application [13]. The electrospinning method creates nanofibers with a high specific surface area, as the electrospun fiber diameters range from tens of nanometers to a few micrometers. This method has been effectively applied for preparing polymer-based nano or microfibers in recent years. Electrospinning technology has numerous applications in energy and environmental sciences. In addition, metal oxide electrospun nanofibers have been used extensively in photovoltaics, sensor technology, catalysis, medicine, fuel cells, hydrogen storage, and supercapacitors [14,15,16].

Alternative sources of energy have been considered to address climate change and the limited supply of fossil fuels. In the fuel cell, hydrogen (H_2_) burns cleanly and produces no environmental pollutants, unlike the burning of fossil fuels [17,18]. Hydrogen fuel cell applications are a promising technology. In addition, H_2_ has the highest energy density per unit weight as compared with other fuels (i.e., 120.7 kJ g^−1^) [19,20]. Nowadays, steam reforming is the primary method used to create industrial hydrogen. Methanol can be reformed at a lower temperature, with a higher H_2_/C ratio than ethanol. Additionally, methanol lacks a carbon-carbon bond, has low sulfur content (5 ppm), and produces less carbonaceous products. Typically, steam reforming of methanol (SRM), partial oxidation, and decomposition processes are used to make hydrogen from methanol. Because of a low reaction temperature, appropriate water miscibility, high ratio of hydrogen concentration, and low CO level of the SRM process, methanol has attracted a lot of attention as a means of manufacturing hydrogen. Furthermore, SRM is a straightforward, effective endothermic reaction, making it appropriate for use in fuel cell applications [21,22].

The three main processes for producing hydrogen from methanol are (1) partial oxidation, (2) decomposition, and (3) the MSR process. The partial oxidation process only produces 66% of the hydrogen needed for fuel cells and involves an exothermic reaction as well. The highly endothermic reaction and high CO content ratio of the breakdown conversion process make it unsuitable for fuel cell construction. Therefore, the MSR process, which has an endothermic reaction, has been used for hydrogen production, which requires a low reaction temperature to achieve a high hydrogen concentration ratio, a high hydrogen production rate of up to 75%, and a low CO level as a byproduct [23,24].

CH_3_OH → CO + 2H_2_ ΔH_0_ = 92.0 kJ/mol (1)

CH_3_OH + H_2_O → CO_2_ + 3H_2_ ΔH_0_ = 49.5 kJ/mol (2)

CH_3_OH + ½O_2_ → CO_2_ + 2H_2_ ΔH_0_ = −155 kJ/mol (3)

In the MSR process, three reactions typically result in the production of hydrogen from methanol: (4) decomposition of methanol, (5) reformation of methanol steam, and (6) reaction of water gas shift (WGS), as shown below.

CH_3_OH → CO + 2H_2_ (4)

CH_3_OH + H_2_O → CO_2_ + 3H_2_ (5)

CO + H_2_O → CO_2_ + H_2_ (6)

Our research team focuses on the copper-based delafossite materials used in the study for maximum H_2_ production rate in the MSR process. We have previously prepared CuCr_0.4_Fe_0.6_O_2_, CuFeO_2_-CeO_2_, CuCrO_2_, CuFeO_2_ powder, and CuYO_2_ powder for application in the MSR process. We have learned from these catalyst studies that the surface area and catalytic activity of copper-based delafossite materials are crucial to the MSR process and contribute to its high production rate more than other catalysts do [25,26,27].

In this study, the electrospinning method was employed to create the CuYO_2_ nanofiber catalyst, which was then used for MSR. The CuYO_2_ nanofibers created thus far have a delafossite crystal structure. For Cu^+^ ions involved in d–d transitions, the CuYO_2_ fiber demonstrates higher hydrogen generation. It also exhibits a Cu^+^ inter-configuration transition from 3d^9^4s^1^ to 3d^10^. Additionally, in linear coordination, Y^3+^ ions share edges with Cu^+^ ions at the CuYO_2_ octahedral sites. One of the primary reasons why these catalysts are used in the study of hydrogen energy is that the CuYO_2_ nanofiber catalyst has intercalating anionic species [28,29]. During the MSR process, the CuYO_2_ nanofiber exhibited a high rate of hydrogen production and a low rate of coke formation. Additionally, the ability of the CuYO_2_ nanofiber to produce hydrogen at a higher rate than CuFeO_2_ nanopowder, CuCrO_2_ nanopowder, CuCrO_2_ bulk powder, and commercial catalysts (Cu/Al/Zn) at various temperatures will be evaluated.

## 2. Materials and Methods

### 2.1. Materials and Instrumentation

Copper nitrate (99% purity), Yttrium nitrate (99.8% purity), and N, N-dimethylformamide (99.8% purity) were purchased from Aldrich AVATOR chemical company, Taipei, Taiwan. Polyvinyl pyrrolidone (PVP, Mw = 1,300,000) and ethanol were obtained from Echo Chemical Co., Ltd., Miaoli, Taiwan. The material structural properties were identified with a transmission electron microscope (TEM, JEM-2100F, JEOL, Tokyo, Japan). The crystal structure arrangements were characterized with an X-ray diffractometer (XRD) (D2 Phaser, Bruker, Billerica, MA, USA). Field emission scanning electron microscopy (FE-SEM) studies were performed with a JEOL JSM-7610F, Tokyo, Japan. A gas chromatograph (GC 1000, China Chromatography, New Taipei City, Taiwan) with one column (60/80 Carboxen^®^ 1000, SUPER CHROMA ENTERPRISE LTD., Taipei, Taiwan) for H_2_ (7′ × 1/16″ in, stainless steel) was used to measure hydrogen production performance. The thermal decomposition behavior of electrospun fibers was studied by thermogravimetric analysis/differential scanning calorimeter (TGA/DSC, STA 449 F5, NETZSCH, Selb, Germany). The surface area of the CuYO_2_ nanofibers was calculated with a Brunauer-Emmett-Teller (BET) surface area analyzer (TriStar II 3030, Micromeritics Instrument Corporation, Norcross, GA, USA). The adsorbate in the BET surface area studies was high-purity nitrogen gas, and the N_2_ gas volume was measured using P/P_0_ = 0–0.3 at various pressures. The CuCrO_2_-CeO_2_ nanopowders were degassed before BET studies by purging with N_2_ at 150 °C for 12 h.

### 2.2. Preparation of Cu_2_Y_2_O_5_ Nanofibers

The electrospinning precursor was prepared by dissolving 0.548 g of copper nitrate and 0.827 g of yttrium nitrate in 14.4 mL of N, N-dimethylformamide. Then, 2.2 g of polyvinylpyrrolidone (PVP, Mw = 1,300,000) were dissolved in the solution. After 6 h of stirring, an ocean-green, viscous, gel-like precursor solution was obtained. A horizontal syringe pump was loaded with the Al_2_O_3_ precursor solution. Standard electrospinning setups have a high-voltage source connected to a metallic needle, which is connected to a syringe pump. In this study, the syringe pump was connected to a Teflon tube (125 mm in length and 4.2 mm in diameter). The precursor solution was placed in a 10 mL syringe fitted with a 0.5 mm stainless steel needle during the electrospinning process. A voltage of 20 kV was applied to the stainless steel needle tip, and the collector was placed 15 cm away from the needle tip, with the flow set to 0.02 mL/h and temperature maintained at 40 °C. For the formation of Al_2_O_3_ precursor fibers, the electrospun Al_2_O_3_ precursor was distributed uniformly over the collector. Additionally, humidity was controlled below 20% to obtain the as-spun fiber. The as-spun fiber was annealed at 800 °C at a heating rate of 10 °C/min to remove the PVP contained in the as-spun fiber and to synthesize the copper and yttrium into Cu_2_Y_2_O_5_ to facilitate the formation of subsequent CuYO_2_ nanofibers. The prepared Cu_2_Y_2_O_5_ and CuYO_2_ nanofibers were analyzed by TGA, XRD, SEM, TEM, and BET characterization studies.

### 2.3. Methanol Steam Reforming

**Scheme 1:**
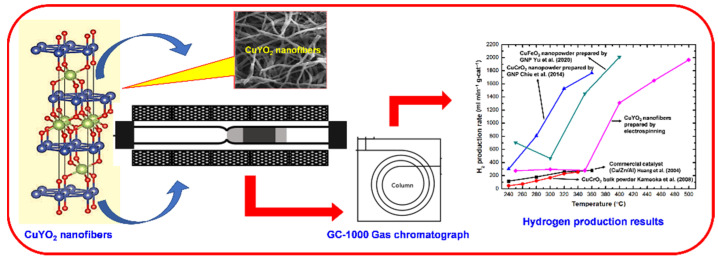
Schematic diagram of the steam reforming process over CuYO_2_ nanofibers.

The methanol steam reforming process was executed in a tube flow reactor which was performed with China Chromatography, New Taipei City, Taiwan. The experiment used a quartz tube with an inside diameter of 1.2 cm and a length of 25 cm as a tube flow reactor for the catalytic reaction. For this, 20 mg of CuYO_2_ nanofibers were sandwiched between the quartz cotton in the middle of the quartz tube and then heated to 250, 300, 350, 400, 450, and 500 °C. Methanol and water were uniformly mixed at a molar ratio of 3:1 to prepare a methanol–water mixture, and that mixture was heated to 80 °C on a hot plate to generate methanol–water vapor by evaporation. Nitrogen was introduced as the carrier gas at a flow rate of 30 sccm. The system was connected to a gas chromatograph, which was used to analyze the gas produced at each temperature for later averaging (Scheme 1).

## 3. Results and Analysis

### 3.1. TGA Analysis of Cu_2_Y_2_O_5_ Nanofibers

**Figure 1:**
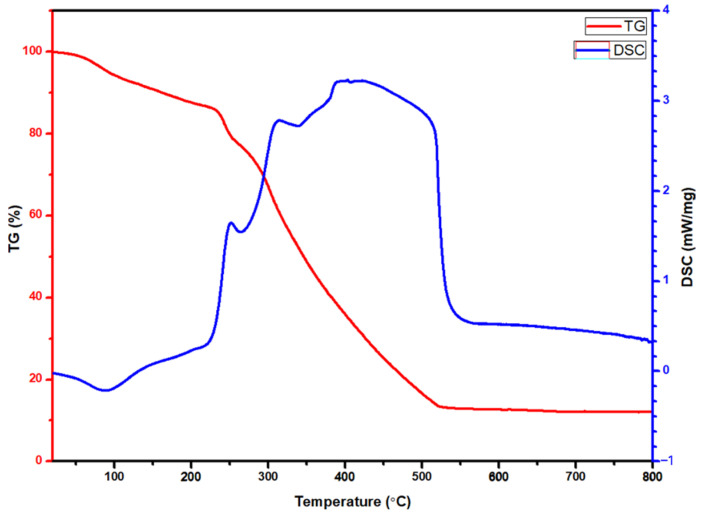
TGA/DSC curves of electrospun Cu_2_Y_2_O_5_ nanofibers.

To observe the decomposition mechanism of the as-spun fibers at high temperatures, a simultaneous thermogravimetric analyzer was used to observe their TGA/DSC curves, and the temperature of the air atmosphere was increased to 800 °C at a heating rate of 10 °C/min. The TGA/DSC curves of electrospun Cu_2_Y_2_O_5_ nanofibers are shown in Figure 1. The weight loss before 100 °C was due to the volatilization of the remaining water in the sample. The slight weight loss and exothermic slope at approximately 150 °C were due to DMF decomposition. The endothermic slope at 265 °C indicated the decomposition of copper nitrate to form CuO. From 250 °C to 520 °C, a large amount of heat was generated and the weight was greatly reduced by 85%, which occurred due to the decomposition of PVP. During this decomposition, the endothermic trend at about 340 °C indicated the complete removal of water by Cu_2_Y_2_O_5_ nanofibers. The endothermic change at around 360 °C represented the decomposition of yttrium nitrate to form Y_2_O_3_. The endothermic slope at 700 °C indicated the combination of Y_2_O_3_ and CuO to form Cu_2_Y_2_O_5_.

### 3.2. XRD Analysis of Cu_2_Y_2_O_5_ Nanofibers and CuYO_2_ Nanofibers

**Figure 2:**
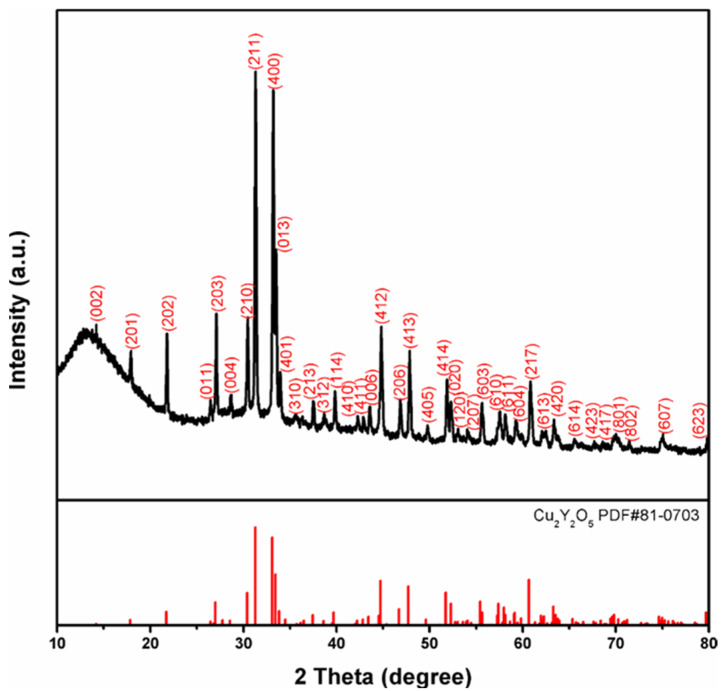
XRD pattern of Cu_2_Y_2_O_5_ nanofibers.

**Figure 3:**
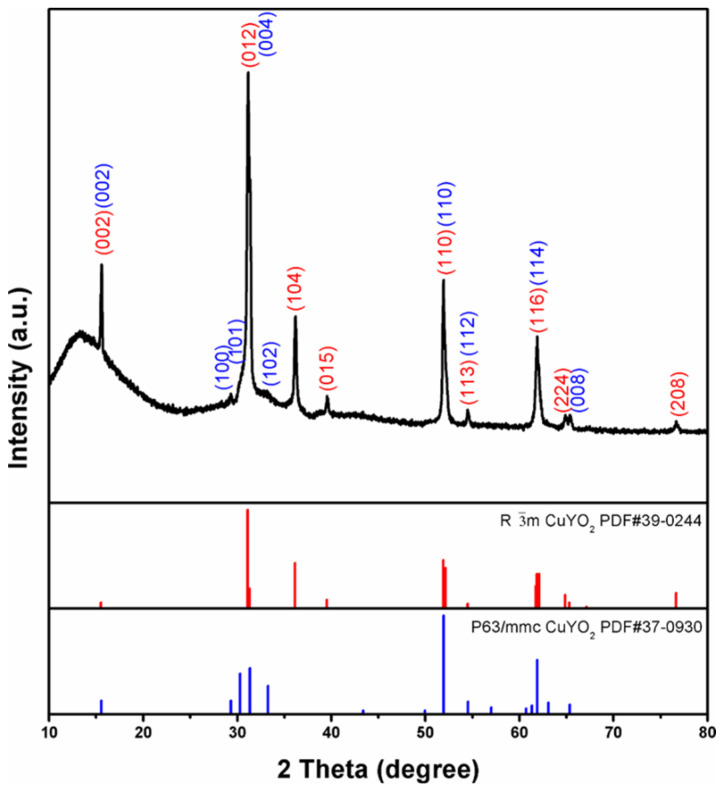
XRD pattern CuYO_2_ nanofibers.

The XRD pattern of Cu_2_Y_2_O_5_ nanofibers is shown in Figure 2. After the as-spun fibers were annealed at high temperatures, the copper nitrate and yttrium nitrate decomposed and formed copper yttrium oxide. According to JCPDS card (Joint Committee on Powder Diffraction Standards) PDF#81-0703, pure Cu_2_Y_2_O_5_ is an orthorhombic phase. The orthorhombic Cu_2_Y_2_O_5_ is a minor phase in the mixture of cubic Y_2_O_3_ and CuO. The XRD pattern of CuYO_2_ nanofibers is shown in Figure 3. After Cu_2_Y_2_O_5_ fibers were annealed at 780 °C in a nitrogen atmosphere, the Cu_2_Y_2_O_5_ was deoxygenated to form CuYO_2_. According to JCPDS cards PDF#39-0244 and PDF#37-0930, CuYO_2_ is composed of rhombohedral and hexagonal phases, respectively.

### 3.3. FESEM and TEM Analyses of Cu_2_Y_2_O_5_ Nanofibers

**Figure 4:**
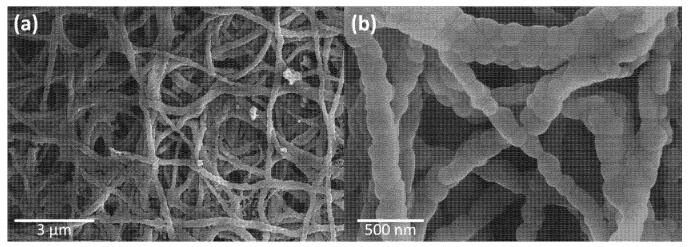
FESEM image of Cu_2_Y_2_O_5_ nanofibers with different magnification (**a**) ×10.0 k and (**b**) ×50.0 k.

**Figure 5:**
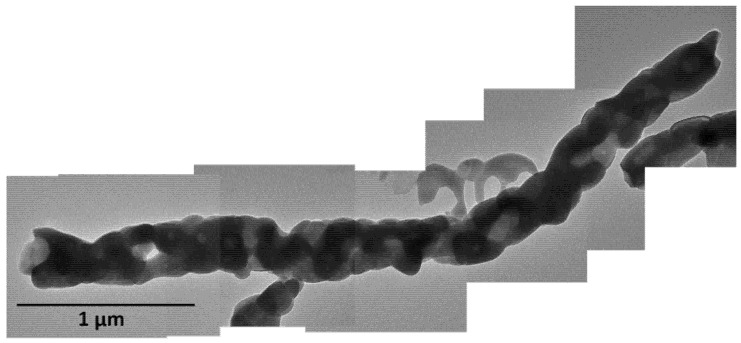
Merged TEM image of Cu_2_Y_2_O_5_ nanofibers.

Figure 4a,b shows the FESEM image of the Cu_2_Y_2_O_5_ nanofibers. Based on PVP, the average fiber diameter was about 950 ± 27.6 nm. The morphology of the as-spun fibers after they were annealed in air at a heating rate of 10 °C/min to 800 °C formed Cu_2_Y_2_O_5_ nanofibers. In the FESEM image, it could be observed that the surfaces of the Cu_2_Y_2_O_5_ nanofibers were rugged and composed of crystalline particles. Furthermore, the PVP disappeared and the diameters decreased to about 243 ± 22.5 nm because of the high temperature. A TEM image of the Cu_2_Y_2_O_5_ nanofibers is shown in Figure 5. It can be observed from the TEM image that the nanofibers were composed of a series of crystal grains and were about 237 ± 16.9 nm in diameter.

### 3.4. FESEM and TEM Analysis of CuYO_2_ Nanofibers

**Figure 6:**
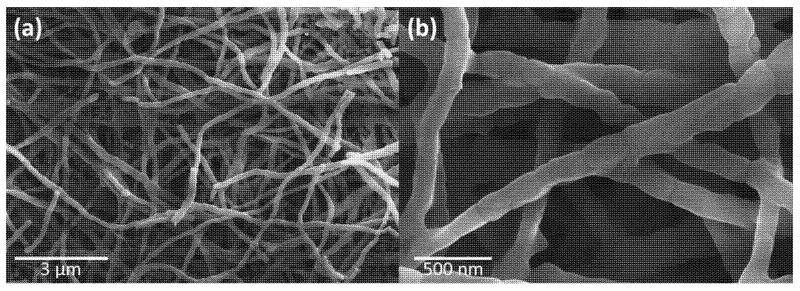
FESEM images of CuYO_2_ nanofibers with different magnification (**a**) ×10.0 k and (**b**) ×50.0 k.

Figure 6a,b presents the FESEM images of CuYO_2_ nanofibers. After Cu_2_Y_2_O_5_ nanofibers were heated at 10 °C/min to 780 degrees and annealed at that temperature for 30 min in a nitrogen atmosphere, they were deoxidized to form CuYO_2_ nanofibers. In the FESEM images, it can be observed that the surfaces of CuYO_2_ nanofibers were smoother than those of Cu_2_Y_2_O_5_ nanofibers.

**Figure 7:**
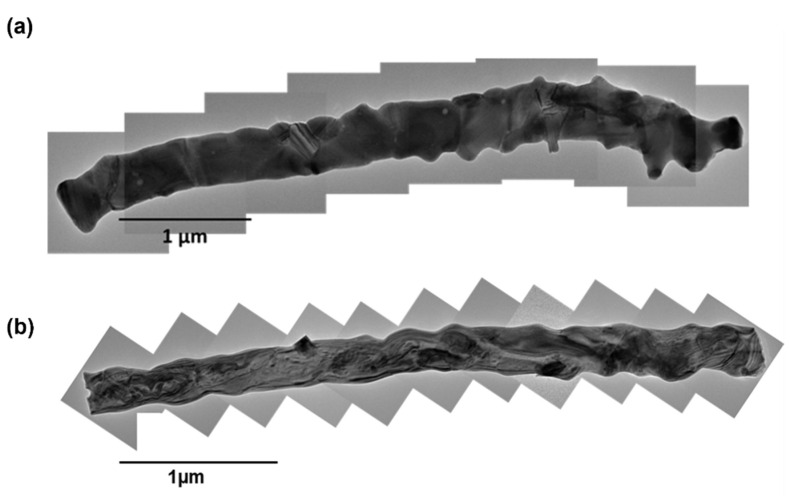
(**a**) Merged TEM image of R$\bar{3}$m CuYO_2_ nanofibers, (**b**) merged TEM image of P63/mmc CuYO_2_ nanofibers.

**Figure 8:**
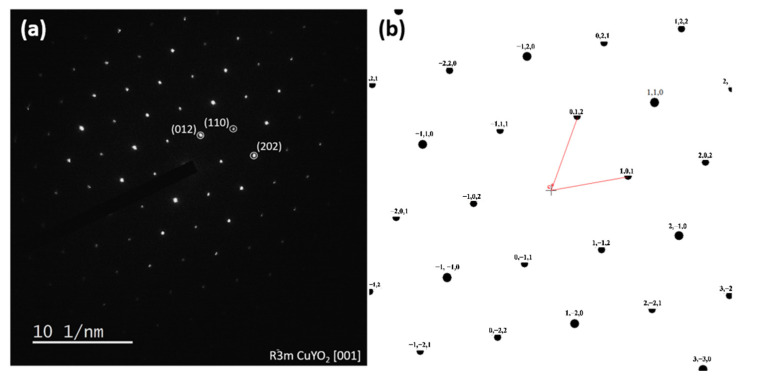
(**a**) SAED pattern of R$\bar{3}$m CuYO_2_ nanofibers, (**b**) simulated diffraction pattern of R$\bar{3}$m CuYO_2_.

**Figure 9:**
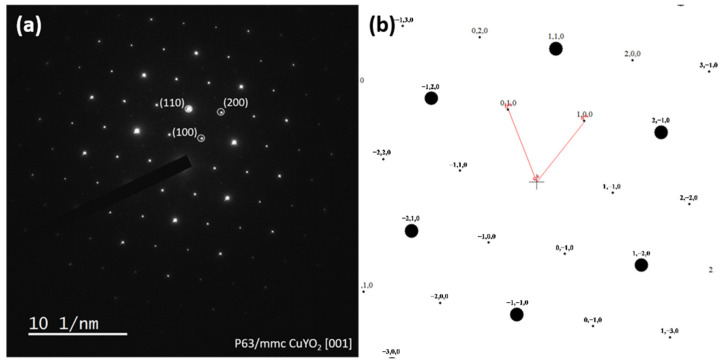
(**a**) SAED pattern of P63/mmc CuYO_2_ nanofibers, (**b**) simulated diffraction pattern of P63/mmc CuYO_2_.

Figure 7a shows a TEM image of an R$\bar{3}$m CuYO_2_ nanofiber, and Figure 7b shows a TEM image of a P63/MMC CuYO_2_ nanofiber. It can be observed from the TEM image that the nanofiber was composed of a series of crystal grains, and the diameter was fixed at 220 ± 17.4 nm. The TEM image SAED patterns are shown in Figure 8a and Figure 9a. The SAED pattern was used to confirm that the nanofibers were composed of CuYO_2_ in two different space groups. From the TEM image, it can be observed that Cu_2_Y_2_O_5_ nanofibers grains merged after annealing to form CuYO_2_ nanofibers. The CuYO_2_ nanofiber grains in the R$\bar{3}$m space group were arranged in a nodular structure, whereas the those in the P63/mmc space group had no obvious grain boundaries. The simulated diffraction pattern of R$\bar{3}$m CuYO_2_ nanofibers is shown in Figure 8b, and the simulated diffraction pattern of P63/mmc CuYO_2_ nanofibers is shown in Figure 9b. Both diffraction patterns confirmed that the d-spacing was consistent with the diffraction patterns of CuYO_2_ nanofibers in the R$\bar{3}$m (The Inorganic Crystal Structure Database, ICSD ID: 60848) and P63/mmc (ICSD ID: 35580) space groups simulated in CaRIne.

### 3.5. Specific Surface Area Analysis of CuYO_2_ Nanofibers

The specific surface areas of the CuYO_2_ nanofibers are listed in Table 1. Table 1 shows the specific surface areas of the different delafossite complexes prepared by glycine-nitrate combustion, solid-state reaction, and electrospinning. The specific surface area of the CuYO_2_ nanofibers produced in this experiment was 10.22 m^2^/g, which is much larger than the specific surface areas of solid-state reactions and other electrospinning products.

### 3.6. Methanol Steam Reforming Performance

**Figure 10:**
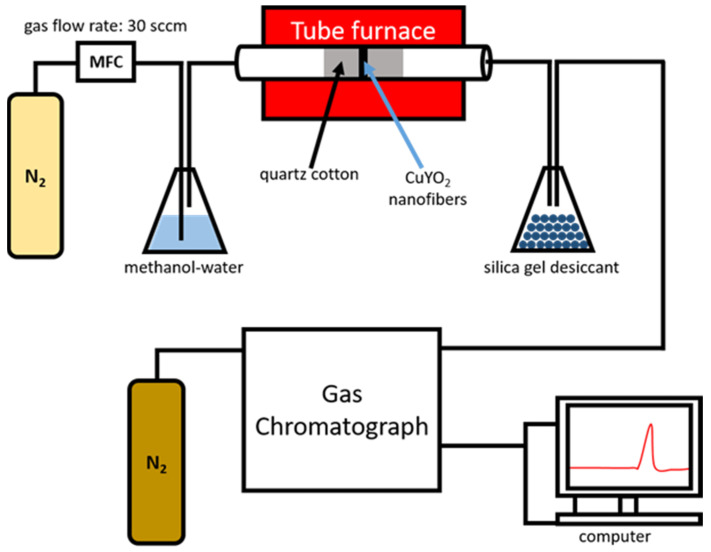
Schematic diagram of the steam reforming of methanol.

The H_2_ production rate with the CuYO_2_ nanofiber catalyst was determined using a gas chromatograph equipped with a thermal conductivity detector. At 250–500 °C, the hydrogen efficiency and productivity at a flow rate of 30 sccm were measured and converted to milliliters per minute (mL STP per min per g-cat) (Figure 10). The temperature of the methanol and water was 80 °C, and the methanol-to-water ratio was 1:4 due to the vapor pressure difference. To maximize yield, the CuYO_2_ nanofiber catalyst powder was activated for 10 min at each temperature without the methanol steam. Then the carrier gas was activated to fill the system with methanol steam, and the hydrogen production performance was measured at the gas outlet pipe. The H_2_ production performances and H_2_ production rates were also calculated with the following Equations (7) and (8) [35,36].

(7) $$\mathrm{Methanol}\;\mathrm{conversion}\;(\%)=\frac{\left(\mathrm{methanol}\right)\;\mathrm{in}\;-\left(\mathrm{methanol}\right)\;\mathrm{out}}{\left(\mathrm{methanol}\right)\;\mathrm{in}}\;\times \;100$$

(8) $$\mathrm{Hydrogen}\;\mathrm{production}\;\mathrm{rate}=\frac{\mathrm{Hydrogen}\;\%.\;\mathrm{mL}.30\;\mathrm{mL}/\min}{{\mathrm{cm}}^{3}\cdot g\;}$$

**Figure 11:**
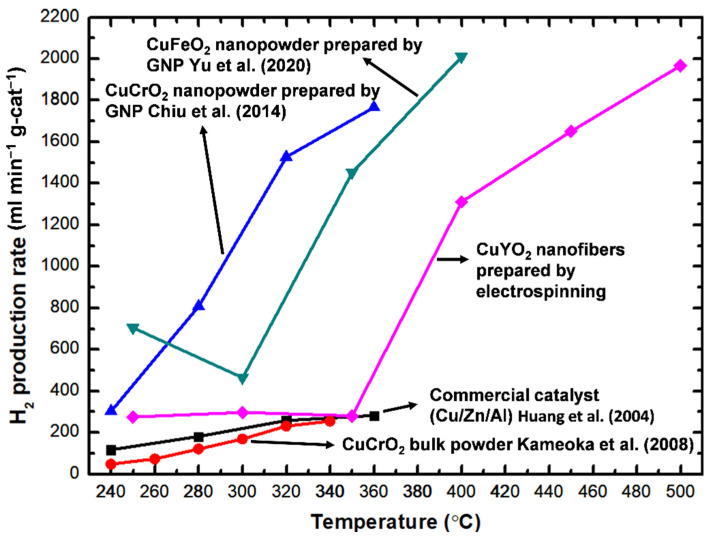
Hydrogen production results of CuYO_2_ nanofibers (electrospun), CuCrO_2_ (GNP) [29], CuFeO_2_ (GNP) [37], CuCrO_2_ (solid-state method) [38], and commercial catalysts [27].

Figure 11 depicts the relationship between the rate of H_2_ production and the reaction temperature in the methanol steam reforming process using the CuYO_2_ nanofiber catalyst. At 400 °C, the maximum hydrogen production rate was 1967.89 mL min^−1^ g-cat^−1^. Table 2 shows the hydrogen production rate of CuYO_2_ nanofibers at different temperatures. The data show that, when the temperature rose, the rate of hydrogen production increased as well. However, when the temperature was too high, the catalyst reached its activity more quickly, and the experiment was not continued at higher temperatures. Figure 11 shows that the electrospun CuYO_2_ nanofibers had much higher hydrogen production efficiency than those of the commercial Cu/Al/Zn catalyst, CuCrO_2_ bulk catalyst, CuFeO_2_ (GNP), CuCrO_2_ (GNP), and CuCrO_2_ (solid-state method) [27,29,37,38]. In addition, the H_2_-activated catalyst is typically dangerous in air because of its high activity and potential for ignition and explosion. In contrast, CuYO_2_ nanofibers are extremely stable in the air. As a result, there is no need for high-temperature activation treatment of the CuYO_2_ nanofiber catalyst for the SRM process. This finding implies that, if CuYO_2_ nanofibers are used in fuel cell vehicles, they can achieve higher efficiency than conventional catalysts. The catalyst stability, SRM conditions, and reactor conditions will be optimized and tested in future work.

### 3.7. XRD and SEM Analysis of CuYO_2_ Nanofibers after MSR

**Figure 12:**
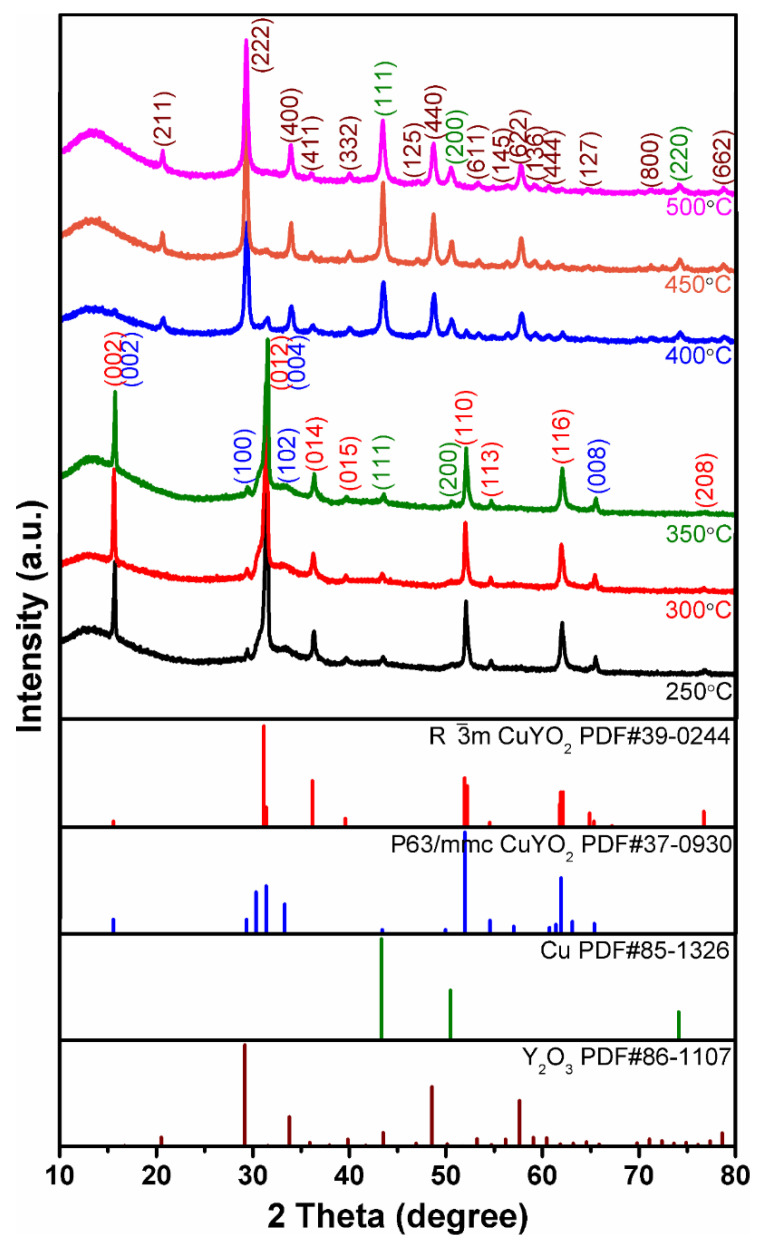
XRD pattern of CuYO_2_ nanofibers after MSR at different temperatures.

Figure 12 shows the XRD pattern of CuYO_2_ nanofibers after MSR at different temperatures (250–500 °C). From the XRD pattern, it was observed that the CuYO_2_ in the R$\bar{3}$m and P63/MMC space groups still maintained a catalytic temperature between 250 °C and 350 °C. After the SRM process, the peaks of the copper (111), (200), and (220) planes could be observed at 2θ = 43.316°, 50.448°, and 74.124° (PDF#85-1326), respectively, caused by the precipitation of CuYO_2_. When the catalytic temperature rose above 400 °C, the peak of CuYO_2_ gradually disappeared and Y_2_O_3_ (PDF#86-1107) began to precipitate. Finally, when the catalytic temperature reached 500 °C, the CuYO_2_ nanofibers almost completely disappeared. In this form, CuYO_2_ nanofibers become the Cu/Y_2_O_3_ phase, increasing both the catalytic activity of Cu and the hydrogen production performance.

**Figure 13:**
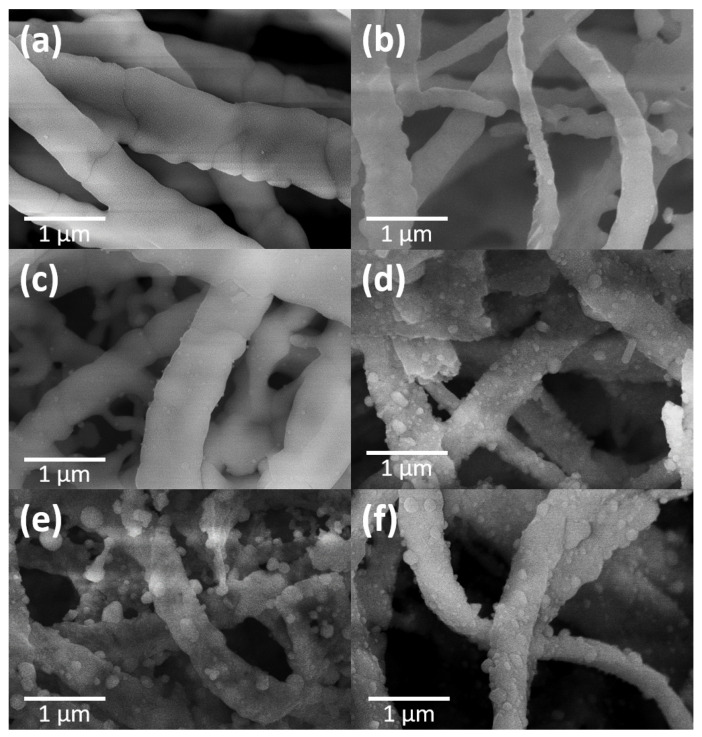
SEM images of CuYO_2_ nanofibers after MSR at different temperatures (**a**) 250 °C, (**b**) 300 °C, (**c**) 350 °C, (**d**) 400 °C, (**e**) 450 °C and (**f**) 500 °C.

Figure 13a–f shows SEM images of CuYO_2_ after MSR at different temperatures. As shown in Figure 13a–c, a few precipitates formed on the surfaces of CuYO_2_ nanofibers after catalysis at a lower temperature. Figure 13d–f illustrates that when the catalytic temperature reached 400 °C, precipitate particles formed on the surfaces of fibers, but these particles did not affect the catalytic activity.

## 4. Conclusions

In this work, Cu_2_Y_2_O_5_ and CuYO_2_ nanofiber catalysts were successfully prepared by electrospinning and applied to the methanol steam reforming process (SRM). The CuYO_2_ nanofibers were studied by scanning electron microscopy, transmission electron microscopy, X-ray diffractometric, Thermogravimetric analysis, and Burner Emmett-Teller analysis. The diameter of the CuYO_2_ nanofibers was fixed at 220 ± 17.4 nm and the specific surface area was 10.22 m^2^/g. In the SRM process, the CuYO_2_ nanofiber catalyst exhibited the best hydrogen production rate of 1967.89 mL min^−1^ g-cat^−1^ at a reaction temperature of 500 °C. Furthermore, under the same operating conditions, the hydrogen generation rate of CuYO_2_ nanofibers was compared with those of previously-reported catalysts. A higher rate of hydrogen production was achieved because of the higher catalytic activity and larger surface area of the CuYO_2_ nanofibers. The catalyst stability and reactor parameters have also been optimized. Based on the H_2_ production performance, the CuYO_2_ nanofibers are appropriate for use as a catalyst for industrial H_2_ production and fuel cell applications in automobiles.

## Figures and Tables

**Table 1:** Specific surface areas of the delafossite complexes prepared by various processes.

Processes	Specific Surface Area (m^2^/g)	Reference
GNP method (CuCrO_2)_	25.70	[30]
GNP method (CuFeO_2)_	11.38	[31]
Solid-state (CuCrO_2_)	1.94	[32]
Solid-state (CuFeO_2_)	0.57	[32]
Electrospinning (CuCrO_2_)	7.85	[33]
Electrospinning (CuFeO_2_)	4.33	[34]
Electrospinning (CuYO_2_)	10.22	This study

**Table 2:** Hydrogen production rates of CuYO_2_ nanofibers at different temperatures.

Temperature (°C)	Hydrogen Production Rate (mL min^−1^ g-cat^−1^)
250	275.61
300	297.77
350	281.17
400	1311.86
450	1651.84
500	1967.89

## Data Availability

Not applicable.
